# Relations Between Early Neuromuscular Alterations, Gastrointestinal Dysfunction, and Clinical Nutrition in Critically Ill Patients: An Exploratory Single-center Cohort Study

**DOI:** 10.1007/s12028-020-00960-0

**Published:** 2020-04-03

**Authors:** Felix Klawitter, Johannes Ehler, Daniel A. Reuter, Robert Patejdl

**Affiliations:** 1 Department of Anaesthesiology and Intensive Care Medicine, University Medical Center Rostock, 18057 Rostock, Germany; 2 Oscar Langendorff Institute of Physiology, University Medical Center Rostock, Gertrudenstraße 9, 18057 Rostock, Germany

## Introduction

Several pathophysiological conditions are known to promote alterations of gastrointestinal (GI) motility and malnutrition in the critically ill [1]. Among these are lesions of the central, the autonomic, the enteric, and the somatic nervous system. Nevertheless, rather few studies have addressed the hypothetical interdependence between neurological and GI complications in the critically ill [2, 3].

Patients with critical illness neuromyopathy (CINM) are commonly exposed to risk factors of GI dysfunction [4]. We hypothesize that CINM and intensive care unit-acquired weakness (ICUAW) might be independent risk factors for GI dysmotility in critically ill patients.

## Methods

**Fig. 1:**
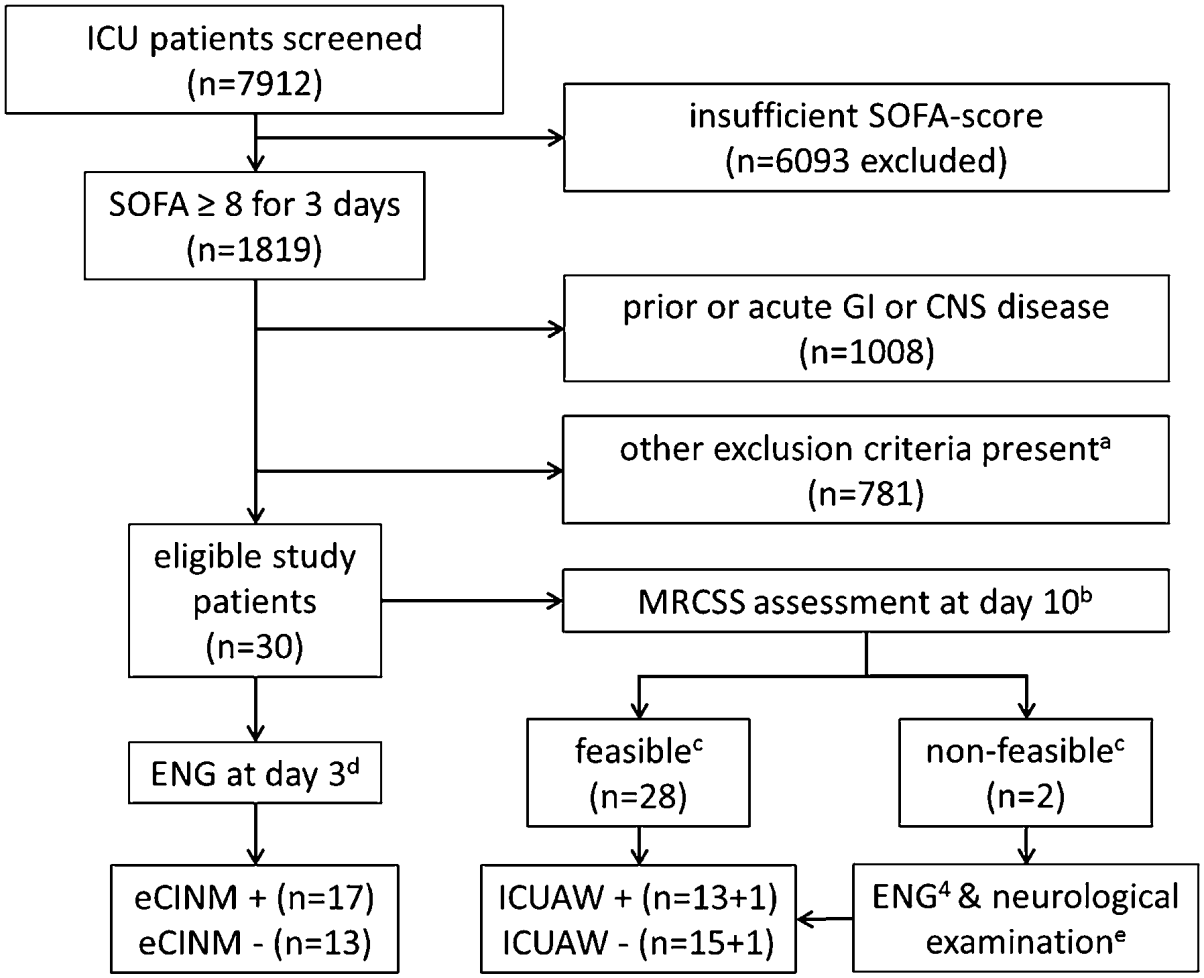
Selection of patient sample and classification of patients as eCINM± and ICUAW± . a: Exclusion criteria were: any pre-existing neuromuscular impairment or gastrointestinal disease of any kind, gastrointestinal surgery prior to ICU admission or within the following 14 days after admission to the ICU, high-dose glucocorticoid treatment (i.e., more than 300 mg of methylprednisolone or equivalent doses of other steroids), prior treatment at another ICU for more than 24 h, missing informed consent by the patient or a legal representative, participation in another clinical trial or expected imminent death. b: MRCSS is assessed by summing up strength scores graded 0–5 from each of 3 muscle groups of each extremity tested by instructing the patient to forcefully abduct the shoulder, flex the elbow, extend the wrist, flex the hip, extend the knee, and dorsiflex the foot. c: Eligibility for MRCSS testing was stated when a patient had to follow standardized requests: “Open and close your eyes”; “Look at me”; “Open your mouth and put out your tongue”; “Nod your head”; “Raise your eyebrows until I have counted to five”. d: see “methods” section for applied electrophysiological criteria of eCINM. e: Whenever MRCSS testing was not possible, patients were also classified ICUAW on day 10 if neurological examination revealed a symmetric flaccid palsy and a loss of deep tendon reflexes in all tested limb muscles

Data were derived from a current prospective observational study including patients ≥ 18 years with a sequential organ failure assessment (SOFA) score ≥ 8 on three consecutive days within the first five days after intensive care unit (ICU) admission by analyzing medical reports for parameters related to nutrition and GI function (ClinicalTrials.gov: NCT02706314, local ethics board identifier A 2016-0016). The study protocol is summarized in Fig. 1.

### Assessment of Early Critical Illness Neuromyopathy (eCINM) and Intensive Care Unit: acquired Weakness (ICUAW)

On study day 3 and 10, electroneurography (ENG) was performed by recording compound motor action potentials (CMAP) from the abductor digiti minimi (ADM) and the extensor digitorum brevis (EDB) muscles and sensory nerve action potentials (SNAP) from radial and sural nerves. A CINM-typical alteration was stated when CMAP amplitude was below 4 mV and SNAP amplitude was smaller than 7.5 µV for the radial and 10 µV for the sural nerve. When less than four of the recording sites gave amplitudes above the stated cutoff values, the patient was classified early CINM (eCINM) positive. ICUAW was diagnosed if the Medical Research Council sum score (MRCSS) was < 48 points [5]. When MRCSS could not be assessed the ICUAW status was stated based upon neurological examination and second ENG (Fig. 1).

### Assessment of GI function and Clinical Nutrition

Over 14 days, we analyzed data on swallowing function assessed by a fiberoptic endoscopic swallowing evaluation (FEES), peroral nutrient supplementation, the extent of calories delivered via a feeding tube and the duration of feeding tube dependence, the gastric residual volume (GRV), and the frequency of bowel movements. To estimate confounding effects, plasma glucose concentration, and administered doses of opioids, laxatives and prokinetic drugs were analyzed. Measurements of GRV were standardized according to local nursing guidelines with nasogastric tubes all of the same type and diameter.

### Statistics

Statistics were done with MS-Excel 2010 (Microsoft, Redmond, WA, USA) and IBM SPSS Statistics (version 25, Chicago, IL, USA). According to the distribution of data (using Shapiro–Wilk test), Student’s *t* test or Mann–Whitney test was used for continuous variables. Chi-square test or Fisher’s exact test was used for categorical variables. Kendall’s *τ* was calculated in bivariate correlations. For nonparametric analysis, we used partial rank correlations, and for two variables, a confounder corrected *τ* (*τ*_confounder_) was calculated. Statistical significance was considered at *p* < 0.05.

## Results

### Patient Demographics and Baseline Parameters

From 7912 patients screened initially, 30 of those (28 surgical and 2 non-surgical) that had been enrolled in the original trial also met the additional criteria of this study (Fig. 1). At day 3, 17 patients were classified eCINM+ and 13 eCINM− according to ENG findings. With regard to the MRCSS at day 10 and the neurological examination, 16 patients were classified ICUAW+ and 14 ICUAW−. Irrespective of the classification, the different study groups were comparable in demographics and clinical baseline characteristics listed in Table 1, except for higher initial APACHE II, SOFA, and mNUTRIC scores in the group of patients classified as ICUAW+ at day 10.

**Table 1:** Study population characteristics and surrogate parameters of clinical nutrition and gastrointestinal function

Parameters	ICUAW+	ICUAW−	*P* value	eCINM+	eCINM−	*p* value
Study population characteristics						
Patient number (*n*)	14	16	N/A	17	13	N/A
MRCSS day 10 (*n* = 28)	29.3 ± 13.0	54.9 ± 4.6	** < 0.001**	40.6 ± 13.7	46.2 ± 18.8	0.38
Age (years)	68.2 ± 11.3	59.8 ± 15.5	0.19	67.2 ± 11.9	66.8 ± 15.9	0.94
Female	7/14	6/16	0.49	9/17	2/13	0.06
APACHE II	26.1 ± 3.5	22.9 ± 6.2	0.15	24.5 ± 5.6	24.2 ± 4.7	0.85
SOFA-score day 3	13.7 ± 3.1	9.4 ± 1.6	** < 0.001**	12.4 ± 3.4	10.2 ± 2.1	0.07
SOFA-score day 10	6.6 ± 3.2	3.9 ± 3.2	**0.004**	5.4 ± 3.3	4.8 ± 3.2	0.64
Patients with diabetes mellitus (*n*)	4/14	4/16	0.83	3/17	5/13	0.24
Surrogate parameters of clinical nutrition and gastrointestinal function						
Total observation period (days)	196	222	N/A	238	182	N/A
mNUTRIC score	7 ± 0.9	6 ± 1.6	**0.03**	4.8 ± 0.9	4.5 ± 0.9	0.39
Confirmed swallowing disorder** (*n*)	6/14	0/16	**0.005**	4/17	2/13	0.98
Days with nasogastric tube in situ (n)	160 (82%)	80 (36%)	** < 0.001**	143 (60%)	97 (53%)	0.16
Days with oral intake (n)	18 (9%)	117 (52%)	** < 0.001**	64 (27%)	71 (39%)	**0.014**
Patients receiving normal diet after 14 days (n)	0/14	6/16	**0.019**	2/17	4/13	0.19
Mean energy via feeding tube (kcal/day)*	1022 ± 777	205 ± 383	** < 0.001**	897 ± 734	830 ± 725	0.48
Days with GRV* (*n*)	100 (63%)	52 (65%)	0.78	99 (41%)	53 (29%)	**0.01**
Mean GRV per day (ml) *	157 ± 266	167 ± 240	0.78	194 ± 286	112 ± 189	**0.008**
Days with bowel movements (*n*)	82 (41%)	91 (43%)	0.92	108 (45%)	65 (36%)	0.06
Days with mechanical ventilation (*n*)	176 (89%)	104 (47%)	** < 0.001**	182 (76%)	98 (54%)	** < 0.001**
Days with nasogastric tube in situ (*n*)	143 (81%)	48 (46%)	** < 0.001**	139 (76%)	80 (82%)	0.37
Days with oral intake (*n*)	14 (8%)	29 (28%)	** < 0.001**	28 (15%)	15 (15%)	0.99
Mean energy via feeding tube (kcal/day)*	1017 ± 779	415 ± 470	** < 0.001**	906 ± 742	888 ± 722	0.85
Days with GRV* (*n*)	94 (61%)	18 (49%)	0.2	96 (53%)	47 (48%)	0.45
Mean GRV per day (ml)*	162 ± 267	181 ± 248	0.62	197 ± 289	117 ± 200	**0.03**
Days with bowel movements (*n*)	73 (41%)	45 (43%)	0.8	83 (46%)	35 (36%)	0.13
Days with endotracheal intubation	98 (50%)	50 (23%)	** < 0.001**	93 (39%)	55 (30%)	0.06
Days with Nasogastric tube in situ (*n*)	79 (81%)	35 (70%)	0.15	69 (74%)	45 (82%)	0.29
Mean energy via feeding tube (kcal/day)*	707 ± 688	358 ± 388	**0.01**	621 ± 677	596 ± 558	0.83
Days with GRV* (*n*)	46 (47%)	23 (46%)	0.92	41 (44%)	28 (51%)	0.42
Mean GRV per day (ml)*	94 ± 146	167 ± 254	0.14	121 ± 192	109 ± 183	0.84
Days with bowel movements (*n*)	28 (29%)	7 (14%)	**0.03**	21 (55%)	35 (64%)	** < 0.001**

Bold values indicate statistical significance

Data presented as sum or mean ± SD. Parameters indicated with “*” are assessed only for days at which patients were supplied with a feeding tube. **Number of patients with FEES-confirmed diagnosis of swallowing disorder. Patients were tested when were compliant, able to sit upright and follow specific commands

*APACHE II* acute physiology and chronic health evaluation score II as assessed at ICU admission, *eCINM* early critical illness neuromyopathy, *GRV* gastric residual volume, *ICUAW* intensive care unit-acquired weakness, *mNUTRIC* modified nutrition risk in critically ill score, *MRCSS* Medical Research Council sum score, *SOFA* sequential organ failure assessment score

### Clinical Nutrition, Motility, Laxative and Prokinetic Treatment in Relation to Neuromuscular Dysfunction

**Fig. 2:**
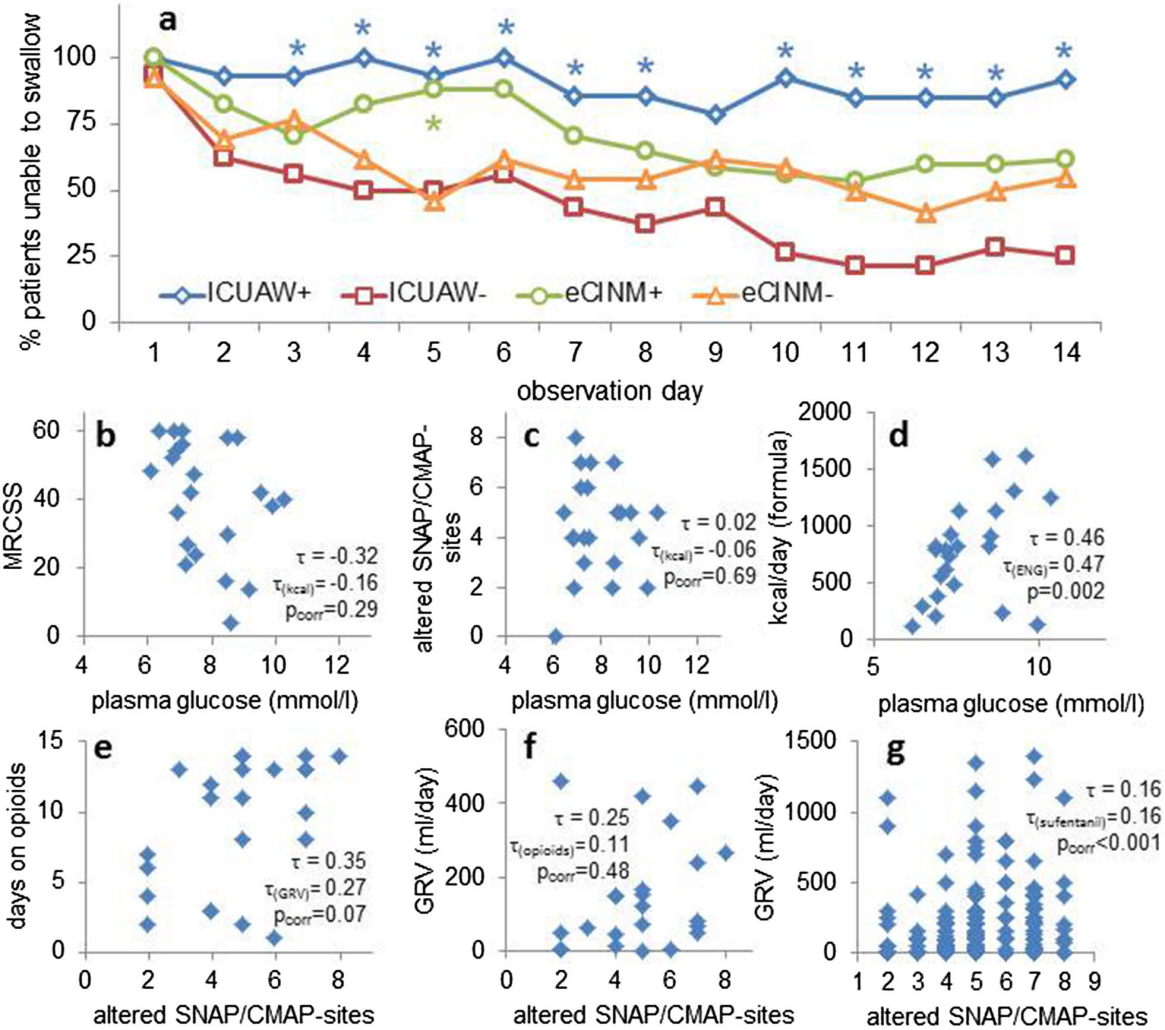
**a** Changes in the percentage of patients receiving oral alimentation. The sample was divided into groups either based upon the results of muscle strength on day 10 (ICUAW ±) or depending on the results of an ENG on day 3 (CINM ±). Dots depict mean values calculated from the subsamples. Asterisks indicate significance of difference between respective groups at a level of *p* < 0.05. The patient numbers in each group are given in Table 1, line 1. eCINM: early critical illness neuromyopathy. ICUAW: intensive care unit-acquired weakness. **b–d** Correlations of mean daily plasma glucose levels of patients with MRCSS (**b**), the number of altered SNAP/CMAP sites in ENG (**c**) and enteral energy delivery (*d*, *n* = 22 for all). Kendall’s tau (*τ*) for the correlation of plasma glucose with MRCSS is > 0.299 and thus per se significant. When corrected for energy delivered via the feeding tube, the correlation is not significant anymore. In the figures, “*p*_corr_” depicts *p* value after correction for delivered formula energy. **e–g** Correlations of the number of altered muscle/nerve recording sites on day 3 with overall opioid treatment (**e**), with GRV calculated as mean value of all GRV measured in single patients over the whole observation period (**f**) and with all single GRV values measured on sufentanil-treatment days (**g**). For mean calculations of GRV, only days when patients were supplied with a feeding tube were calculated. In the figures, “*p*_corr_” depicts *p* value after correction for GRV (**e**), for the number of days patients were on opioids (**f**), and for the administered daily dose of sufentanil (**g**). *CMAP* compound motor action potential. *MRCSS* Medical Research Council sum score. *SNAP* sensory nerve action potential

Results with regard to nutrition and GI motility are listed in Table 1. For all formed groups, the mNUTRIC score was > 4 and significantly higher in ICUAW+ patients, indicating high-risk for malnutrition. Concordantly, within 14 days both ICUAW+ and eCINM+ patients had fewer days with oral intake. Peroral alimentation was significantly reduced for almost all study days in ICUAW+ patients and at study day 5 in eCINM+ patients (Fig. 2a). Patients with ICUAW were longer dependent on nasogastric tubes and received more calories via tube feeding. Furthermore, none of the ICUAW+ patients received normal diet by day 14.

Mean daily GRV differed significantly in relation to the eCINM status. In ICUAW− patients, GRV was higher within the first seven days, whereas it tended to be lower in this subgroup from day 10 onwards. In contrast, mean GRV in eCINM+ patients was almost constantly equal or higher than in eCINM− patients. By individually analyzing days on mechanical ventilation, significantly more ICUAW+ patients needed nasogastric tube feeding than patients without ICUAW and the GRV was still higher in eCINM+ patients. Bowel movements were more frequently detected in ICUAW+ patients, but reduced in eCINM+ patients by analyzing days with intubation.

The duration of laxative treatment with lactulose and sodium picosulfate was significantly higher in the ICUAW+ group (lactulose: given on 63% of observation days in ICUAW+ patients vs. 40% in ICUAW−; sodium picosulfate: ICUAW+ : 57%, ICUAW−: 34%, *p* < 0.001 for both).

### Plasma Glucose, Opioids, and Narcotics in Relation To Neuromuscular Dysfunction

Mean daily plasma glucose concentrations were significantly higher in ICUAW+ and eCINM+ patients. The MRCSS, but not the number of ENG alterations, correlated significantly with the mean plasma glucose, but missed statistical significance when corrected for enteral nutrition in partial correlations (Fig. 2b–d).

ICUAW+ and eCINM+ patients received opioids more frequently, but there was no difference in the treatment duration with sufentanil (most frequently administered opioid on 37% of all days). Patients without eCINM tended to receive sufentanil for a longer period. Administered sufentanil doses were significantly lower in ICUAW+  and eCINM+ patients.

Neither bivariate nor partial correlations (corrected for GRV) between the mean number of days at which patients received opioids and the number of altered SNAP/CMAP sites reached significance (Fig. 2e). The same applied to the correlation between mean GRV and ENG alterations (corrected for days with opioids, Fig. 2f). In contrast, single-day GRV was correlated with the number of the patients’ ENG alterations, even if corrected for sufentanil effects (Fig. 2g). There were no group differences in administered doses and application times of other opioids or narcotics.

## Discussion

The results of this study indicate that alterations in electrophysiological parameters may be predictive for increased GRV, prolonged feeding tube dependence, less frequent bowel movements in intubated patients, prolonged opioid prescription, and decreased sufentanil-dose demands in the later course of critical illness. When ICUAW status was assessable by day 10, ICUAW+ subjects had retrospectively suffered from more severe disease, had more often required mechanical ventilation, and had shown a prolonged dependency on tube feeding and opioids. Considering all this and the fact that baseline SOFA and APACHE II scores were worse in this same group of patients, it seems likely that ICUAW merely reflects foregoing disease severity, whereas early CINM may predict upcoming gastrointestinal signs and symptoms independently from disease severity.

Since hyperglycemia is stated as a risk factor for ICUAW [6], increased glucose levels may either be a cause for or a consequence of ICUAW+ or eCINM+ status. As neither the MRCSS nor the number of ENG alterations was independently correlated with glucose levels (Fig. 2b, c), both findings do not support a causative role of hyperglycemia for the development of ICUAW or eCINM here.

Surprisingly, sufentanil doses were lower in ICUAW+ and eCINM+ patients by comparing days on mechanical ventilation. We hypothesize that the impairment of neuromuscular function influenced the clinical perception of opioid needs due to sensory impairment or muscular weakness with reduced pain-indicating movements. Despite our efforts to compensate opioid effects, we cannot rule out that some of the observed differences in GI function parameters are confounded by opioid exposure.

This study has some relevant limitations: First, the low number of patients resulted from our intended preselection of patients who were diseased so severely that GI and neuromuscular complications were likely to occur. This selection caused the small sample size, which limits the certainty of all stated statistical relations. Second, some of the observed effects were rather small. But although more sensitive functional tests or biomarkers might have yielded more specific results, it was our genuine interest to analyze the impact of ICUAW and eCINM on clinically accessible and relevant parameters. Third, our criteria to define eCINM were based on our monocentric reference values and differ from general definitions suggested by other groups. The decision to define own criteria was motivated by the fact that there are no generally applicable criteria for diagnosing eCINM in a cohort like ours [5].

The preliminary results of this exploratory study need verification in future studies. These may exclusively enroll critically ill surgical patients and combine the essential early electroneurographic assessment with more sensitive markers of GI function (e.g., sonographic or refractometric assessment of gastric transport) or measurement of biomarkers of nutrition and intestinal failure (e.g., prealbumin, citrulline, fatty acid-binding protein) [7]. Furthermore, we conclude that the concept of ICUAW is not suitable to study the potential relevance of neuromuscular impairment for early gastrointestinal complications of critical illness.
